# Forkhead Box O (FOXO) signaling in NSCLC: pathways to targeted therapies

**DOI:** 10.17179/excli2024-7272

**Published:** 2024-05-31

**Authors:** Lakshmi Thangavelu, Terezinha de Jesus Andreoli Pinto, Sachchidanand Pathak, Abhishek Tiwari, Varsha Tiwari, Gaurav Gupta, Kumud Pant, Saurabh Gupta, Moyad Shahwan

**Affiliations:** 1 Centre for Global Health Research, Saveetha Medical College, Saveetha Institute of Medical and Technical Sciences, Saveetha University, India; 2 Department of Pharmacy, Faculty of Pharmaceutical Sciences, University of Sao Paulo, Professor Lineu Prestes Street, Sao Paulo 05508-000, Brazil; 3 Kashi Institute of Pharmacy, Mirzamurad, Varanasi, India; 4 Pharmacy Academy, IFTM University, Lodhipur-Rajput, Moradabad, UP, 244102, India; 5 Chitkara College of Pharmacy, Chitkara University, Rajpura, Chandigarh, India; 6 Centre of Medical and Bio-allied Health Sciences Research, Ajman University, Ajman, UAE; 7 Graphic Era (Deemed to be University) Clement Town Dehradun- 248002, India; 8 Graphic Era Hill University Clement Town Dehradun, 248002, India; 9 Chameli Devi Institute of Pharmacy, Indore, Madhya Pradesh, India

## ⁯⁯⁯

Cellular signaling pathways form a complex network crucial for the pathogenesis of most types of cancer, including lung cancer, which remains one of the most death-causing neoplasms worldwide. The Forkhead Box O signaling pathway has received considerable investigation due to its role in cell cycle regulation, apoptosis, DNA repair, and the response to oxidative stress (Abdelfatah et al., 2019[1]; Al-Tamari et al., 2018[2]). Recent investigations have proven the critical functions of FOXO proteins, particularly FOXO1 and FOXO3a, in lung carcinogenesis, opening prospects for using one of its components as a target for developing antitumor approaches. Cisplatin is the most commonly applied first-line chemotherapeutic agent for treating non-small-cell lung cancer (Chen et al., 2022[3]). The drug has been shown to interact with the FOXO signaling pathway, which induces cancer cell death by apoptosis. The authors showed that cisplatin treatment induced the expression and nuclear translocation of FOXO1 and FOXO3a in NSCLC cells, making them susceptible to cisplatin-mediated apoptosis. Inhibition of FOXO1 and FOXO3a considerably decreased the susceptibility of those cells to the antineoplasm, both *in vitro* and *in vivo*, demonstrating the central function of these FOXOs in cisplatin cytotoxic action. This discovery indicates that targeting FOXO1 and FOXO3a might improve the efficacy of cisplatin-based NSCLC therapy (Gupta et al., 2017[4]).

In addition, β-Elemene, a chemical with an anti-cancer effect, exhibited inhibitory lung cancer cell growth activity through regulation of the FOXO3a signaling pathway. It reduces Stat3 phosphorylation and miRNA155-5p mRNA and increases FOXO3a and IGFBP1 expressions. This inactivation of Stat3 creates a feedback loop between miRNA155-5p and FOXO3a, which also partakes in the induction of IGFBP1 expression. This complexity of competing protein expression emphasizes the prospect of FOXO3a signaling pathway point for lung cancer cell growth inhibition, presenting β-Elemene as a potential therapeutic due to its mechanism of action (Sun et al., 2020[5]). Another aspect of NSCLC genetic expression complexity is the repression of DNMT3B transcription by FOXO3a and its overexpression by MDM2. This successful up-regulation of the DNMT3B gene, out of the complex relationship with both FOXO3a and MDM2, results in poor prognosis for lung cancer patients. Therefore, targeted inhibition of the FOXO3a/DNMT3B/MDM2 axis might be seen as a new therapeutic direction for lung cancer treatment, making the existence of the epigenetic linkage with this pathology evident. The FOXO signaling pathway is vital in lung cancer cell resistance to drugs. FoxO3a participates in a negative feedback mechanism of the EGFR signaling pathway through the downregulation of EPS8, decreasing NSCLC aggressiveness and gefitinib resistance. That could imply targeting the FoxO3a-EPS8 axis as a hopeful approach in therapy, for it shows potential NFCLC resistance overcoming power (Wen et al., 2019[6]).

Finally, environmental factors, including exposure to airborne particulate matter, have also been connected to the formation of lung cancer pathogenesis by modulating the activation of the FOXO signal. PM10 exposure inhibited apoptosis in lung epithelial cells via the activation of the PI3K/AKT/FoxO3a axis, impacting how environmental pollutants contribute to lung carcinogenesis by allowing cells with defective DNA to survive. The findings imply that a much more comprehensive understanding of lung cancer pathogenesis focused on understanding genetic and environmental components is required to develop successful preventive and therapeutic interventions (Yang et al., 2017[7]). In summary, the FOXO signaling pathway that influences the pathogenesis of lung cancer includes apoptosis, drug sensitivity, resistance and the effects of environmental factors. The investigations examined above provide new knowledge about lung cancer cells' complexity and identify possible therapeutic obstacles to the FOXO signaling pathway. Combined with a joint connection, the investigations shed more light on this course's importance in lung cancer, guiding the next round of potential therapeutic measures to boost patient outcomes in a challenging cancer type.
